# A focus on lucigenin as a potent photoredox catalyst

**DOI:** 10.1039/d6sc90202b

**Published:** 2026-10-29

**Authors:** Daniel Aßenmacher, Robert Hein

**Affiliations:** a Organisch-Chemisches Institut, Universität Münster Corrensstraße 36 48149 Münster Germany robert.hein@uni-muenster.de

## Abstract

Old dog, new tricks: The well-established fluorescent and chemiluminescent probe lucigenin (Luc^2+^, *N*,*N*′-dimethyl-9,9′-bisacridinium), can also act as a powerful photoredox catalyst. Readily commercially available as a nitrate salt, a straightforward anion exchange to the more lipophilic Luc^2+^(BF_4_^−^)_2_ was recently demonstrated by Feringa and coworkers to afford a soluble, versatile catalyst (A. Matei, B. Roure, X. Chen, S. B. Beil and B. L. Feringa, *Chem. Sci.*, 2026, **17**, 11436, https://doi.org/10.1039/d6sc02111e). Due to its high excited-state reduction potential, this dicationic scaffold enabled the oxidative azolation of unactivated arenes with a broad substrate scope.

## Abstract 2

This focus article highlights recent work in which Matei *et al.* (*Chem. Sci.*, 2026, https://doi.org/10.1039/d6sc02111e) repurposed lucigenin, a common fluorescent probe, as a potent photoredox catalyst for the oxidative azolation of arenes.

## Graphical abstract

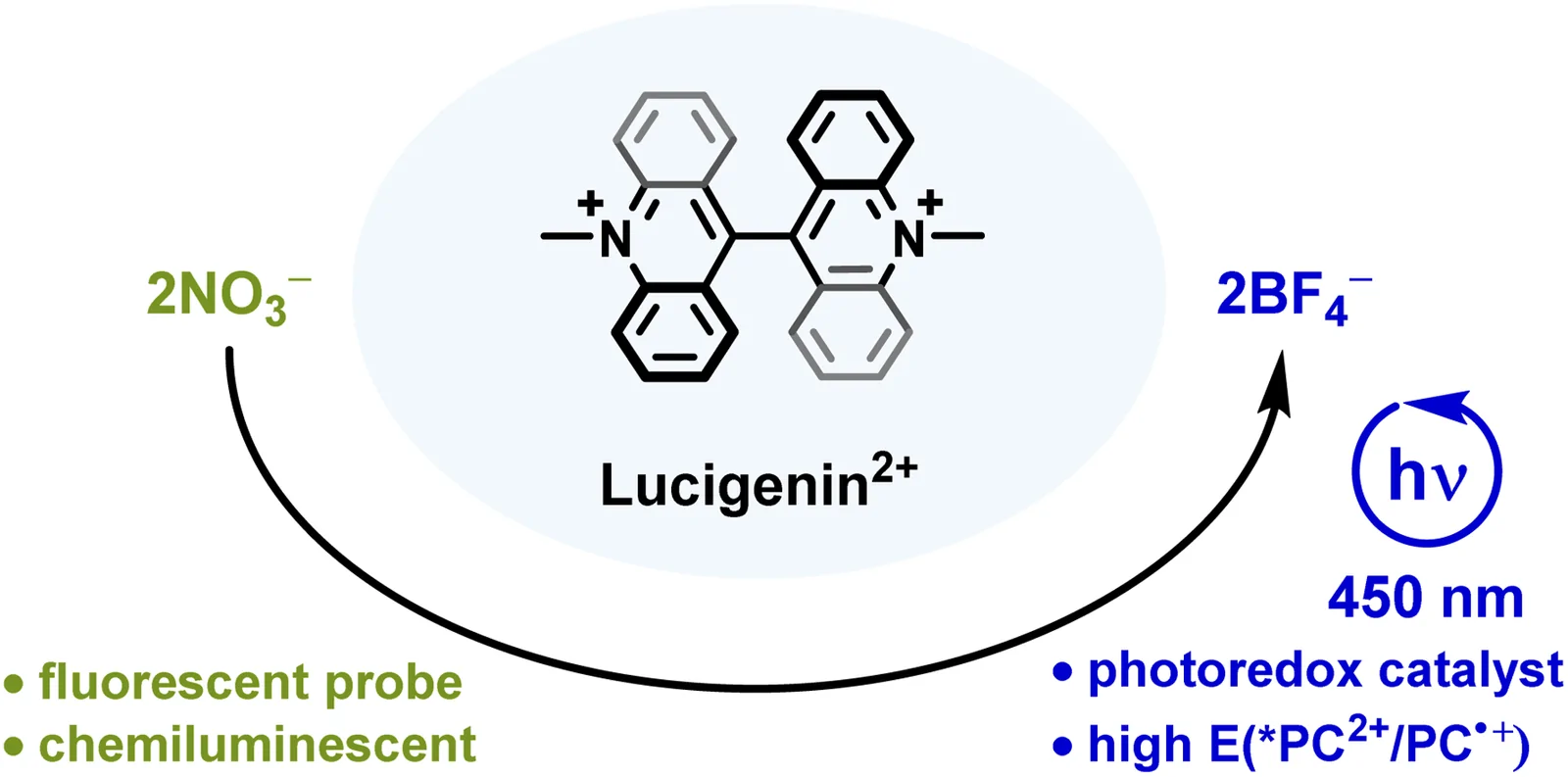

Photoredox catalysis (PRC) has emerged as an increasingly important tool in a wide range of synthetic transformations, supporting new reactivities and reactions under mild conditions. While many different metal-based and organic photoredox catalysts have been developed, a common challenge remains the activation of relatively inert substrates, *i.e.*, those with high redox potentials, such as simple unactivated arenes.^1^

Strategies for overcoming this hurdle include, among others, consecutive photoinduced electron transfer (conPET) and electro-activated photoredox catalysis (e-PRC), wherein highly potent catalysts are generated *in situ* by sequential activation steps.^2^ In this manner, very challenging photoredox transformations can be carried out; however, this often involves highly reactive catalyst intermediates and/or complex experimental setups.

Alternatively, tuning the redox potentials of traditional “single photon” organic photoredox catalysts by synthetic modifications has been the target of many recent studies. A particularly appealing and privileged scaffold in this context is the cationic acridinium motif (Acr^+^). For example, seminal work by Nicewicz and coworkers demonstrated that acridinium photoredox catalysts enable C–H amination of electron-rich arenes.^3^ While many common, “early” acridinium photoredox catalysts, including the Nicewicz catalyst, are potent oxidizers (with typical excited-state reduction potentials *E*(*Acr^+^/Acr˙) in the range of +2.0–+2.15 V *vs.* SCE), they are typically incapable of oxidatively engaging less electron-rich arenes like halogenated arenes or benzene. However, transforming these simple feedstock chemicals would provide a valuable alternative route towards more complex architectures, which in the case of arene aminations are traditionally prepared *via* the Buchwald–Hartwig reaction.

Addressing this problem, Kerzig, Hansmann and co-workers recently reported on dicationic acridinium-carbene hybrid photocatalysts that are inherently electron deficient and possess high excited-state reduction potentials (up to +2.5 V *vs.* SCE). This enabled oxidative azolations of electron deficient arenes as well as benzene.^4^

In a recent study reported in this journal,^5^ Feringa and coworkers employed a similar photoredox approach by using lucigenin (Luc^2+^), a simple dicationic acridinium dimer to catalyse a wide range of azolation reactions (Fig. 1A and B).

**Fig. 1:**
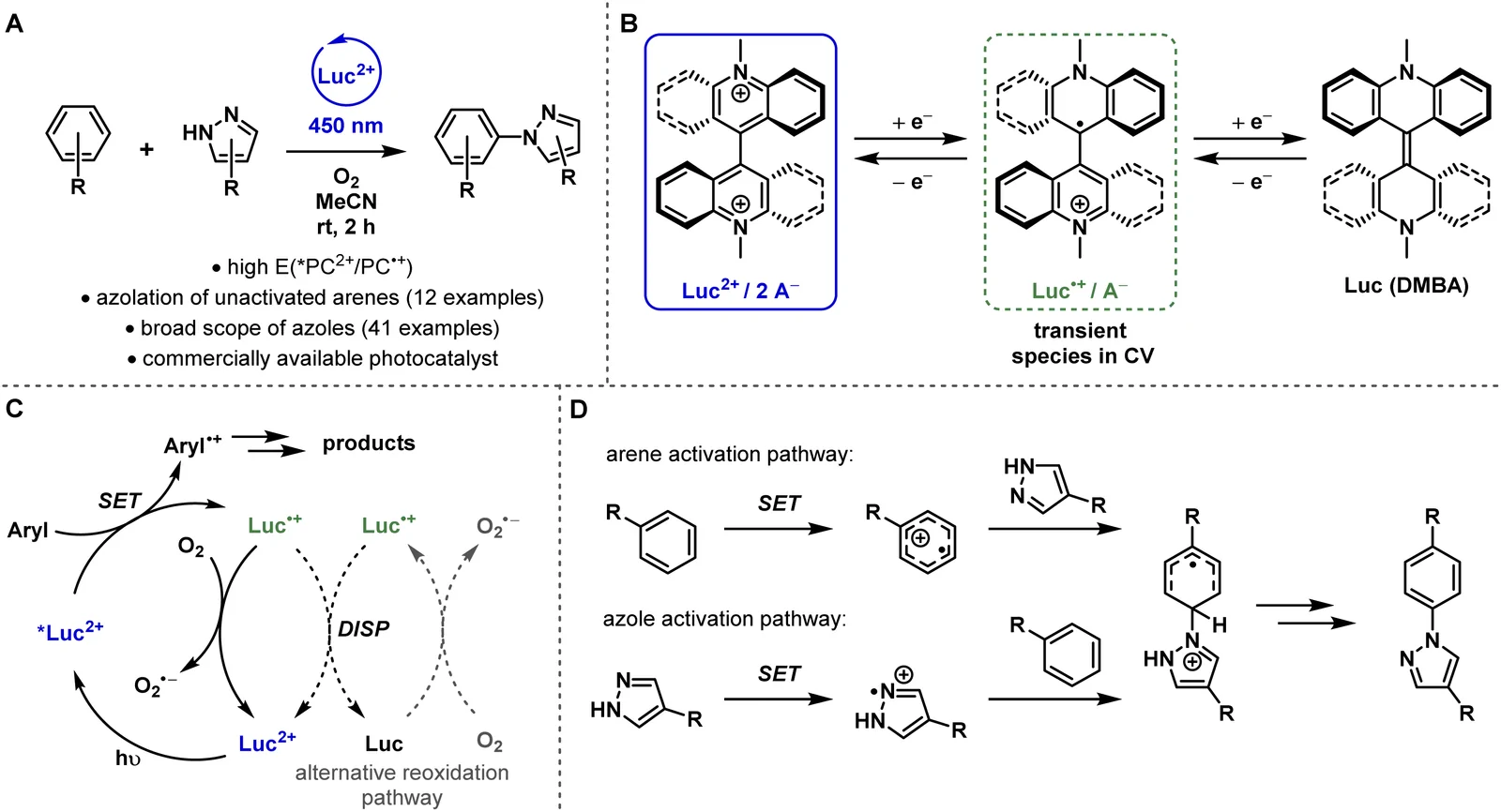
(A) General overview of lucigenin-catalysed photoredox azolation reported by Feringa and coworkers.^5^ (B) Structures and simplified redox reactions of lucigenin. (C) Proposed photocatalytic cycle (solid lines), as well as an alternative catalyst reoxidation pathway *via* disproportionation “DISP” proposed by the authors of this focus article (grey, dashed lines). (D) Arene and azole activation pathways.

This includes the coupling of benzene with a large array of different mono- and multi-substituted pyrazoles, as well as imidazoles, triazoles and tetrazoles, including an intramolecular C–H amination of the tetrazole-based hypertension drug irbesartan. Various *N*-arylated pyrazoles were also accessed by reaction of pyrazole with benzenes, including electron deficient halobenzenes, highlighting the broad reaction scope with respect to substitution at both coupling partners. Furthermore, under the optimised reaction conditions, no additional additives, such as the commonly used fluorinated solvent hexafluoroisopropanol (HFIP, a potentially problematic PFAS), nor additional oxidants (apart from atmospheric oxygen), are required.

However, most notable is the use of lucigenin, perhaps the simplest acridinium dimer, as a catalyst. This dication has been structurally known for at least 120 years^6,7^ and has, as a commercially available nitrate salt, found many applications as an optical (sensor) probe with bright fluorescence and chemiluminescence.^8,9^ While the nitrate salt itself is not an effective photoredox catalyst due to its limited solubility in organic solvents, a simple anion exchange to Luc^2+^(BF_4_^−^)_2_, a bench stable salt, was shown to solve this problem. This represents an elegant and experimentally straightforward entry into simple, yet potent, photoredox catalysts and begs the question of how many other long-known (organic) scaffolds might also be good (photoredox) catalysts.

Employing a range of mechanistic studies, the authors suggest a possible mechanism for the observed reactivity, whereby the photoredox cycle depicted in Fig. 1C (solid lines) generally mirrors those of other acridinium systems. Specifically, the photoexcited catalyst *Luc^2+^ oxidizes the substrate by single electron transfer (SET) and is thereby itself reductively quenched to generate Luc˙^+^. As the estimated excited-state reduction potential of the catalyst *E*(*Luc^2+^/Luc˙^+^) of ∼2.23 V *vs.* SCE is insufficiently high to oxidize some of the electron-deficient benzene derivatives, the typically invoked arene activation pathway (Fig. 1D, top) may not be applicable in all cases. Instead, oxidation of the azole first (Fig. 1D, bottom) was suggested as an alternative route, a proposal that was experimentally supported by, among others, Stern–Volmer quenching experiments.

Depending on the exact substrate combinations and concentrations, as well as reaction conditions, both pathways may contribute towards the overall reactivity, both in this and other studies.

The (photo)redox properties of lucigenin may thus far appear to match those of standard, monoacridinium systems. However, a closer inspection reveals that it displays unique properties brought about by its dimeric structure.

Specifically, as an archetypal overcrowded bistricyclic aromatic ene (BAEs), the neutral doubly-reduced Luc state, also known as dimethylbiacridylene (DMBA), adopts a folded conformation,^10^ whose geometry differs significantly from the dicationic (Luc^2+^) and radical cation (Luc˙^+^) states, which both adopt twisted conformations (Fig. 1B).^11^ As a result, electron transfer is strongly coupled to geometric rearrangements, inducing unique dynamic redox properties.^12–14^ This includes apparent two-electron redox processes for both the oxidation and reduction, which occur at significantly different potentials (hysteresis). In typical voltammograms, the intermediate radical cation (Luc˙^+^) is a species that cannot be observed or generated in noticeable quantities because it is quickly consumed, for example, by a thermodynamically more favourable second electron transfer (potential inversion) or by disproportionation.^11,15^

The latter process may also be relevant in the photoredox cycle discussed above: we, the authors of this focus article, suggest that the disproportionation of the twisted radical cation (Luc˙^+^) can also regenerate the active catalyst Luc^2+^ as well as the neutral Luc (initially in the twisted state, which then quickly rearranges to the folded neutral state, thereby shifting the equilibrium in this direction). Oxygen as the stoichiometric oxidant could then reoxidise the neutral state. Overall, this would result in a reduced steady-state concentration of the potentially reactive/unstable Luc˙^+^ state and may favourably contribute to the catalytic performance of the system.

It should be noted that this additional/alternative reoxidation pathway has no bearing on the mechanistic proposal with respect to the synthetically relevant steps.

Nevertheless, a consideration of the implications of the rare^4^ two-electron redox chemistry may provide useful insights into the performance of these systems and enable potentially novel applications in synthesis and beyond.

Along these lines, a recent study by Slanina and coworkers elegantly showed that pairs of dynamic redox switches (including the Luc^2+^/DMBA system) can undergo bidirectional photoredox-mediated switching.^16^

Due to its straightforward synthesis by dimerization of acridones,^17^ a further structural diversification of the lucigenin scaffold should also be straightforward. Similarly, hybrids^18,19^ with, or homodimers of, related cationic organic photocatalysts/building blocks, such as (seleno)xanthylium^20,21^ may also possess interesting photoredox behaviour.

## Author contributions

D. A. and R. H. wrote the manuscript.

## Conflicts of interest

There are no conflicts to declare.

## Note added after first publication

This article replaces the version published on 29^th^ October 2026. Ref. 4–21 were incorrectly ordered after typesetting and the order has now been corrected. The Royal Society of Chemistry apologises for this error.
